# Effects of White Matter Hyperintensities on Verbal Fluency in Healthy Older Adults and MCI/AD

**DOI:** 10.3389/fnagi.2021.614809

**Published:** 2021-05-06

**Authors:** Alar Kaskikallio, Mira Karrasch, Juha Koikkalainen, Jyrki Lötjönen, Juha O. Rinne, Terhi Tuokkola, Riitta Parkkola, Petra Grönholm-Nyman

**Affiliations:** ^1^Department of Psychology, Åbo Akademi University, Turku, Finland; ^2^Combinostics Ltd., Tampere, Finland; ^3^Turku PET-Centre, University of Turku, Turku, Finland; ^4^Division of Clinical Neurosciences, Turku University Hospital, Turku, Finland; ^5^Department of Radiology, University Hospital of Turku, Turku, Finland

**Keywords:** verbal fluency, white matter hyperintensities, Alzheimer’s disease, mild cognitive impairment, vascular cognitive impairment

## Abstract

**Background:**

White matter hyperintensities (WMHs) are markers for cerebrovascular pathology, which are frequently seen in patients with mild cognitive impairment (MCI) and Alzheimer’s disease (AD). Verbal fluency is often impaired especially in AD, but little research has been conducted concerning the specific effects of WMH on verbal fluency in MCI and AD.

**Objective:**

Our aim was to examine the relationship between WMH and verbal fluency in healthy old age and pathological aging (MCI/AD) using quantified MRI data.

**Methods:**

Measures for semantic and phonemic fluency as well as quantified MRI imaging data from a sample of 42 cognitively healthy older adults and 44 patients with MCI/AD (total *n* = 86) were utilized. Analyses were performed both using the total sample that contained seven left-handed/ambidextrous participants, as well with a sample containing only right-handed participants (*n* = 79) in order to guard against possible confounding effects regarding language lateralization.

**Results:**

After controlling for age and education and adjusting for multiple correction, WMH in the bilateral frontal and parieto-occipital areas as well as the right temporal area were associated with semantic fluency in cognitively healthy and MCI/AD patients but only in the models containing solely right-handed participants.

**Conclusion:**

The results indicate that white matter pathology in both frontal and parieto-occipital cerebral areas may have associations with impaired semantic fluency in right-handed older adults. However, elevated levels of WMH do not seem to be associated with cumulative effects on verbal fluency impairment in patients with MCI or AD. Further studies on the subject are needed.

## Introduction

Aging is often accompanied by vascular changes in cerebral white matter (WM) (Feigin et al., 2003), which typically show up as white matter hyperintensities (WMHs) when magnetic resonance imaging (MRI) is utilized (Pantoni et al., 2007). These cerebrovascular changes can have a variety of effects on cognitive functions, including impairments to information processing speed, executive functions, working memory, episodic memory, as well as linguistic functions (de Groot et al., 2000; Gunning-Dixon and Raz, 2000; Nordahl et al., 2005, 2006; Au et al., 2006; Pantoni et al., 2007; Zhou and Jia, 2009; Chin et al., 2012; Jokinen et al., 2012; Maillard et al., 2012; Lampe et al., 2019).

Cerebrovascular pathology and Alzheimer’s disease (AD) are intertwined in several respects, as both share common risk factors (Duron and Hanon, 2008) and often overlap and co-occur (Toledo et al., 2013). Furthermore, the risk for developing AD is increased by vascular diseases and elevated WMH (Breteler, 2000; Wolf et al., 2000; Prins et al., 2004), whereas AD patients exhibit elevated levels of cerebral WM pathology (Brickman, 2013) as well as degeneration in specific WM tracts (Mito et al., 2018). Thus, it is of critical importance to study the effects of WM pathology on cognition in AD as well as in mild cognitive impairment (MCI), which is often an early stage of AD. However, the topic has received considerably less attention than the association between gray matter morphology and cognition (for exceptions, see Brickman et al., 2008; Brickman, 2013; Ramirez et al., 2014; Bilello et al., 2015; Mito et al., 2018; Kaskikallio et al.,2019a,b).

A deficit that occurs fairly early in AD is impaired word finding (Farrell et al., 2014). Word generation is commonly measured by verbal fluency (VF) tasks that involve generating words according to cues within a preset time interval: category cues are used for semantic fluency and letter cues for phonological fluency (Lezak et al., 2012). Verbal fluency tasks require using a variety of executive control processes (e.g., focusing on the task, updating material, inhibiting irrelevant responses) and are thus also seen as effective probes for executive functioning (Henry and Crawford, 2004). Overall, AD patients appear to exhibit larger impairments in semantic fluency than in phonological fluency (Henry et al., 2004). This likely reflects the deterioration of the semantic memory store traditionally linked to accumulating neuropathological changes in AD (Chertkow and Bub, 1990; Hodges et al., 1992).

Functional neuroimaging studies have indicated that VF tasks rely on relatively left-lateralized cortical networks (Birn et al., 2010), involving the frontal and temporal regions, anterior cingulate, superior parietal cortex, left hippocampus, thalamus, and cerebellum (Phelps et al., 1997; Gourovitch et al., 2000; Abrahams et al., 2003; Costafreda et al., 2006; Robinson et al., 2012; Biesbroek et al., 2016). Furthermore, the right hemisphere has been suggested to be more involved in semantic fluency tasks over phonological fluency tasks in a number of studies (Schlösser et al., 1998; Donnelly et al., 2011; Glikmann-Johnston et al., 2015). More specifically, areas in the left inferior/middle frontal cortex seem to contribute to both types of fluency (Costafreda et al., 2006; Wagner et al., 2014; Schmidt et al., 2019). However, phonological fluency seems to rely relatively more on the left frontal cortex (presumably reflecting the need for additional strategic effort) and semantic fluency relatively more on the left temporal cortex (presumably reflecting the need for retrieval from semantic memory) (Henry and Crawford, 2004; Baldo et al., 2006, 2010). Since phonological tasks require more effort and executive control, they are expected to impose more substantial demands on planning and strategy formation than semantic fluency tasks, which can rely more on utilizing pre-existing semantic networks (Henry and Crawford, 2004). Nonetheless, various retrieval strategies can be used in both types of tasks.

According to the dual stream model, the system for processing auditory speech involves two language streams that diverge from the superior temporal gyrus (Hickok and Poeppel, 2007; Saur et al., 2008). A left-dominant dorsal stream connects the superior temporal lobe and posterior frontal premotor association cortices via the arcuate fasciculus and superior longitudinal fasciculus, facilitating sensorimotor language production. On the other hand, a bilateral ventral language stream connects the superior and middle temporal lobe with the ventrolateral prefrontal cortex via the extreme capsule and the middle/inferior longitudinal fasciculi, extracting meaning from sounds (Hickok and Poeppel, 2007; Saur et al., 2008). The microstructural integrity of WM tracts from both pathways has been associated with VF performance in studies that have included healthy adolescents and adults as well as various clinical populations. These tracts include the left arcuate fasciculus and the bilateral superior longitudinal fasciculus for the dorsal stream (Peters et al., 2012; Allendorfer et al., 2016; Rodríguez-Aranda et al., 2016; Blecher et al., 2019), and the bilateral inferior longitudinal fasciculus for the ventral stream (Allendorfer et al., 2016; Rodríguez-Aranda et al., 2016; Blecher et al., 2019). Associations have also been reported for the bilateral frontal aslant track (Catani et al., 2013; Kinoshita et al., 2015; Blecher et al., 2019) and the corpus callosum (Rodríguez-Aranda et al., 2016).

Although numerous studies have been published on the neuroanatomic correlates of VF, research about the neurocorrelations between WM and VF in MCI and AD populations has been fairly limited. Studies utilizing diffusion tensor imaging have reported associations between semantic fluency and WM microstructure measures in the corpus callosum, right anterior periventricular, and posterior periventricular regions (Kavcic et al., 2008; Chen et al., 2009). Likewise, Rodríguez-Aranda et al. (2016) reported associations between semantic fluency and a bilateral network of WM tracts (uncinate fasciculus, inferior fronto-occipital fasciculus, forceps minor, and corpus callosum) as well as phonological fluency and several left-hemisphere tracts (anterior thalamic radiation, superior longitudinal fasciculus, inferior longitudinal fasciculus). Finally, Serra et al. (2010) reported that no significant associations exist for these groups specifically.

Overall, the research literature regarding the effects of WM pathology on verbal fluency in AD is quite limited, and previous studies have contained fairly small samples. We have previously examined effects of WM pathology on both general cognitive functioning (Kaskikallio et al., 2019a) as well as on specific cognitive domains (Kaskikallio et al., 2019b, 2020) in cognitively healthy adults and patients with MCI or AD. In these studies, verbal fluency was not included in the verbal function domain score (Kaskikallio et al., 2019b, 2020) due to relatively low shared variance with the other verbal tasks in factor analysis—thus supporting the view that VF tasks tap additional cognitive processes such as executive functions (e.g., Henry and Crawford, 2004; Aita et al., 2016). In this study, we investigated verbal fluency *per se.* The aim was to examine the associations between WM pathology and VF in a sample consisting of a group of cognitively healthy older adults and a group of amnestic MCI and AD patients. A special focus was on examining possible group-wise effects, i.e., would there be differences in the effects of WM pathology between cognitively healthy and MCI/AD patients. The sample utilized here is a portion of the sample that has been used previously (Kaskikallio et al., 2019a, 2020), with the quantified MRI being utilized in Kaskikallio et al. (2019b).

## Materials and Methods

### Participants

The data used in the current study were originally collected in the DEMPET and TWINPIB research projects over several years at the National PET-Centre in Turku, Finland (Kemppainen et al., 2006; Koivunen et al., 2011; Scheinin et al., 2011). The current sample is a portion of the one that has been utilized before, albeit with differing cognitive measurements and neuroimaging analysis methods (Kaskikallio et al.,2019a,b, 2020). The studies were carried out in accordance with relevant guidelines and regulations and were approved by the Joint Ethical Committee of the University of Turku and Turku University City Hospital. The participants received oral and written information about the study and gave informed consent.

The Petersen et al. (2001) criteria were used for diagnosing MCI, whereas patients with AD fulfilled the Diagnostic and Statistical Manual of Mental Disorders fourth edition (DSM-IV) criteria for dementia as well as the National Institute of Neurological and Communicative Disorders and Stroke/Alzheimer’s Disease and Related Disorders Association (NINCDS-ADRDA) criteria for probable AD (McKhann et al., 1984). Controlled concomitant metabolic and cardiovascular diseases were allowed, but participants with Type I diabetes were excluded. Furthermore, a minimum score of 25 in the Mini-Mental State Exam was required for inclusion into the cognitively healthy group. Patients with MCI were of the amnestic type, which is typically characterized by episodic memory impairment. The time lag between MRI data acquisition and neuropsychological testing was 1 week, on average, and 2 weeks at the at the most. From the original sample of 148 participants, 62 participants had to be excluded due to insufficient MRI data quality for quantification. The final sample consisted of 42 cognitively healthy adults, 14 patients with MCI, and 30 patients with AD. The MCI and AD subgroups were pooled together into due to relatively small group sizes. Further details can be found in Kaskikallio et al. (2019a).

Demographic characteristics of study participants are reported in Table 1. The cognitively healthy and patient (MCI + AD) groups were similar with regard to age [*t*(84) = –0.463, *p* = 0.645], education (*U* = 855.500, z = –0.653, *p* = 0.514) and gender distribution [χ^2^(2) = 0.385, *p* = 0.535]. However, age and education were kept as covariates, as they traditionally have strong associations with cognitive performance. Furthermore, the patient group had lower Mini-Mental State Exam scores than the cognitively healthy group [*t*(83) = 4.846, *p* < 0.001]. Finally, three participants reported being left-handed and four ambidextrous. As we did not want to limit the sample size any further, it was decided to run the analyses both with and without these participants in order to guard against possible confounding effects regarding language lateralization (e.g., Szaflarski et al., 2002).

**TABLE 1:** Demographic and clinical characteristics of study participants.

	All	Cognitively healthy	Patient group (MCI/AD)
*n*	86	42	44
Women%	41.9%	45.2%	38.6%
Age M (SD), years	71.76 (4.73)	71.52 (5.20)	71.00 (4.40)
MMSE Score M (SD)	25.81 (3.57)	27.50 (1.40)	24.16 (4.24)^*a*^
Right-handed	79	38	41
Left-handed	3	1	2
Ambidextreous	4	3	1
Education level			
Primary school	43	20	23
Vocational school	32	15	17
Upper secondary	2	2	0
Academic degree	9	5	4

**MCI, mild cognitive impairment; AD, Alzheimer’s disease; MMSE, Mini-Mental State Exam. ^*a*^Data are missing from one participant in the patient group.**

### Verbal Fluency Measures

Measures for semantic fluency (animals) and phonological fluency (“S”) were administered. The participants were asked to orally produce as many words as they could for the span of 1 min. The total number of correct responses was reported. Cognitively healthy controls had the best performances in all word fluency measures, although no statistically significant differences were found between the groups (see Table 2).

**TABLE 2:** Word fluency performances in whole sample and in subgroups.

Word fluency measure	All	Cognitively healthy	Patient group (MCI + AD)	Group difference^*a*^
***All participants (n* = *86)***				
Semantic fluency	21.55 (6.62)	22.36 (5.39)	20.77 (7.59)	*p* > *0.05*
Phonological fluency	13.37 (6.51)	14.43 (6.03)	12.36 (6.86)	*p* > *0.05*
***Right-handed only (n* = *79)***				
Semantic fluency	21.67 (6.78)	22.50 (5.59)	20.90 (7.71)	*p* > *0.05*
Phonological fluency	13.38 (6.47)	14.34 (5.93)	12.49 (6.89)	*p* > *0.05*

**MCI, mild cognitive impairment; AD, Alzheimer’s disease. Means are reported first, followed by standard deviations in brackets. ^*a*^Student’s T-test was used to study differences between groups.**

### MRI Acquisition

A 1.5T Philips Intera (Best, the Netherlands) was used for MRI acquisition. White matter hyperintensities were analyzed using three-dimensional (3D) T1 FFE transaxial (TR/TE 25/5, 58 ms; slice thickness, 2 mm; matrix, 512 × 512) and 2D fluid attenuated inversion recovery (FLAIR) coronal (TR/TE, 11,000/140 ms; slice thickness, 5 mm; matrix, 512 × 512) images. The same sequences were applied to the whole sample. White matter hyperintensities were segmented according to the method presented in Wang et al. (2012). This quantified MRI data has been utilized and details reported previously in Kaskikallio et al. (2020). Comparisons between the cognitively healthy and patient subgroups did not yield statistically significant differences in WMH volumes, although the patient groups exhibited systematically higher mean volumes than the cognitively healthy group.

### Statistical Analysis

Several multiple linear regression analyses were performed for testing the main research questions. For each regression model, age and level of education were entered as control variables in step 1, after which a measure for WMH in each anatomical region of interest was added as a dependent in step 2. Semantic fluency or phonological fluency was set as the independent variable for each analysis. Separate analyses were conducted for the eight anatomical regions of interest (left frontal, right frontal, left parieto-occipital, right parieto-occipital, left temporal, right temporal, bilateral frontal, bilateral parieto-occipital).

Analyses including the whole sample were run first, followed by analyses containing only right-handed participants. Type I errors due to multiple testing were controlled by using the Benjamini–Hochsberg procedure (Benjamini and Hochberg, 1995). A false discovery error rate of 0.05 was used to produce adjusted *p*-values for each step 2 predictor variable, against which the original *p*-values were compared against. The procedure was performed to the nine predictor variables for each hypothesis family (semantic fluency/phonological fluency) for both the total sample and the sample containing only right-handed participants. For those regression models that remained significant after correction, further subgroup analyses were performed separately for the control group and the patient group (MCI + AD). The same procedure to guard against multiple hypothesis testing was performed at this stage. Data analysis was done with the IBM SPSS statistics software v. 24.

## Results

Analyses concerning the total sample (controls, MCI/AD) and containing right-handed, ambidextrous, and left-handed participants (n = 86) were performed first, followed by identical analyses performed on a sample containing only right-handed participants (*n* = 79) (see Table 3 for main analyses). Age and education were controlled for in step 1 of each model.

**TABLE 3:** Regression models predicting word fluency performance from white matter hyperintensities.

	All participants (*n* = 86)						Only right-handed (*n* = 79)					
Independent variables	Semantic fluency			Phonological fluency			Semantic fluency			Phonological fluency		
	*R*^2^	*pΔR*^2^	*B* (95% CI)	*R*^2^	*pΔR*^2^	*B* (95% CI)	*R*^2^	*pΔR*^2^	*B* (95% CI)	*R*^2^	*pΔR*^2^	*B* (95% CI)
***Step 1***												
Model 1 (M1): age and education	0.089	0.021		0.211	0.000		0.085	0.034		0.240	0.000	
***Step 2***												
M1 + frontal L WMH	**0.139**	**0.032**	**–0.98 (–1.88, –0.09)**	0.231	0.150	–0.61 (–1.44, 0.26)	**0.146**	**0.024**	–1.13 (–2.11, –0.16)*	0.253	0.253	–0.50 (–1.38, 0.37)
M1 + frontal R WMH	**0.136**	**0.039**	**–0.67 (–1.31, –0.04)**	0.231	0.153	–0.43 (–1.02, 0.16)	**0.141**	**0.030**	–0.78 (–1.49, –0.08)*	0.253	0.248	–0.37 (–1.00, 0.26)
M1 + frontal L + R WMH	**0.139**	**0.033**	**–0.42 (–0.79, –0.04)**	0.232	0.144	–0.26 (–0.61, 0.09)	**0.145**	**0.024**	–0.48 (–0.90, –0.06)*	0.254	0.240	–0.22 (–0.59, 0.15)
M1 + temporal L WMH	0.100	0.329	–0.93 (–0.28, 0.95)	0.215	0.524	–0.56 (**–**2.28, 1.17)	0.100	0.264	–1.12 (–3.10, 0.86)	0.245	0.455	–0.65 (–2.39, 1.08)
M1 + temporal R WMH	0.125	0.071	–1.43 (–2.97, 0.12)	0.222	0.303	–0.75 (–2.19, 0.69)	**0.144**	**0.025**	–2.01 (–3.76, –0.25)*	0.261	0.145	–1.15 (–2.70, 0.41)
M1 + Temporal L + R WMH	0.115	0.130	–0.69 (–1.59, 0.21)	0.219	0.367	–0.38 (–1.21, 0.45)	0.124	0.071	–0.91 (–1.89, 0.08)	0.254	0.235	–0.52 (–1.39, 0.35)
M1 + parieto-occipital L WMH	**0.133**	**0.046**	**–0.72 (–1.42, –0.01)**	0.246	0.057	–0.63 (–1.27, 0.02)	**0.134**	**0.043**	**–0.75 (–1.48, –0.03)**	0.275	0.058	–0.61 (–1.25, 0.02)
M1 + parieto-occipital R WMH	**0.161**	**0.010**	**–0.75 (–1.31, –0.19)**	0.238	0.094	–0.45 (–0.98, 0.08)	**0.168**	**0.008**	–0.80 (–1.38, –0.22)*	0.270	0.084	–0.45 (–0.98, 0.06)
M1 + Parieto-occipital L + R WMH	**0.151**	**0.017**	**–0.39 (–0.71, –0.07)**	0.243	0.068	–0.28 (**–**1.57, 0.02)	**0.155**	**0.015**	–0.42 (–0.75, –0.08)*	0.274	0.064	–0.28 (–0.57, 0.02)

Separate models have been run for each ROI and cognitive variable. In every model, education and age were entered as control variables in step 1 and the volume of white matter intensities in step 2. The amount of explained variance (R^2^) for each model is reported with the corresponding p-value (pΔR^2^) for the difference in explained variance between model 1 and the current model. Coefficients (B) with 95% CIs are also provided. WMH, white matter hyperintensity; L, left; R, right. *These predictors remained significant after correcting for multiple testing. Models that were initially significant (i.e., before correcting for multiple testing) have been bolded.

**FIGURE 1:**
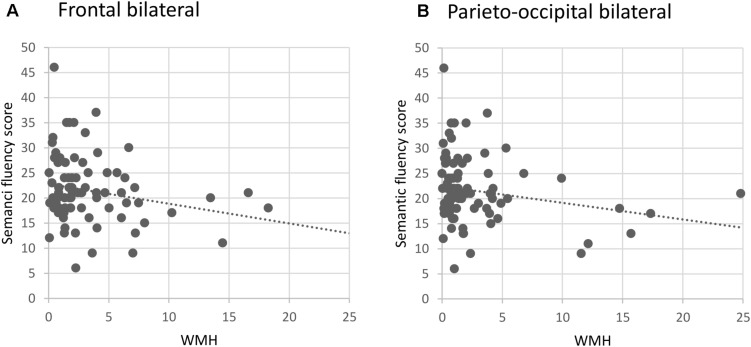
Semantic fluency as a function of bilateral **(A)** frontal and **(B)** parieto-occipital white matter hyperintensities in right-handed participants (*n* = 79). WMH, white matter hyperintensities (ml).

In the whole sample, increased WMH volumes in both the frontal and parieto-occipital areas, bilaterally, were significantly associated with worse performance in the semantic fluency task, although these associations did not survive correction for multiple testing. In the sample containing only right-handed participants, the results were similar, i.e., WMH volumes in frontal and parieto-occipital areas, bilaterally, were associated with lower semantic fluency performance (see Figure 1). Additionally, a significant association was seen between increased WMH volumes in the right temporal lobe and worse performance in the semantic fluency task only in the right-handed participants. The models concerning right-handed participants remained significant after correcting for multiple testing except for the association between left parieto-occipital WMH and semantic fluency.

As only the models concerning right-handed participants survived correction for multiple testing, left-handed/ambidextrous participants were excluded from the follow-up subgroup analyses. These concerned the areas that were significantly associated with semantic fluency after correction (left and right frontal, right parieto-occipital, right temporal) and were run separately for the cognitively healthy and MCI/AD subgroups. However, no significant associations were found concerning these subgroups.

## Discussion

The results indicated that increases in frontal and parieto-occipital WMH volumes, bilaterally, were associated with decreases in semantic fluency when all groups were included (healthy controls + MCI/AD patients). As we preferred to keep the sample size as large as possible, these analyses contained all the participants (including a few left-handed and ambidextrous ones). In order to guard against possible confounding effects regarding language lateralization, additional analyses were performed with only right-handed participants. However, after correcting for multiple testing, only the models concerning the right-handed participants remained significant (all except left parieto-occipital WMH, although bilateral parieto-occipital WMH remained significant). No significant group-specific effects in the control or patient groups specifically were seen.

The indications regarding an association between both frontal and parieto-occipital WMH with decreased semantic fluency are generally in line with previous findings: Verbal fluency performance has been linked with a network of frontal and parietal cortical regions, in addition to the temporal lobe and other subcortical structures such as the anterior cingulate, left hippocampus, thalamus, and cerebellum (Phelps et al., 1997; Gourovitch et al., 2000; Abrahams et al., 2003; Costafreda et al., 2006; Robinson et al., 2012; Biesbroek et al., 2016). However, previous studies have often shown more left lateralized associations for VF, whereas the associations seen here seemed to be bilateral. We would argue that left lateralized networks and certain cortical areas most certainly play a key role in VF tasks but also that VF tasks may rely on a broader bilateral network. This might apply more to semantic fluency, as some investigators have suggested a larger involvement for the right hemisphere in semantic fluency tasks over phonological tasks (Schlösser et al., 1998; Donnelly et al., 2011; Glikmann-Johnston et al., 2015). Indeed, in the current study, right temporal WMH volumes seemed to be associated with decreased semantic fluency performance in right-handed participants.

Related to this, a number of studies on various clinical groups have implicated the right hemisphere in VF tasks: Impaired VF performance has often been reported in patients with right frontal lesions (Perret, 1974; Martin et al., 1990; Loring et al., 1994; Robinson et al., 2012), with a recent study identifying the right inferior frontal gyrus as an important area for semantic fluency (Biesbroek et al., 2016). As for WM tracks, VF impairments have also been associated bilaterally with the inferior fronto-occipital fasciculus and the superior longitudinal fasciculus in MS patients (Blecher et al., 2019) and in a pooled sample of healthy old adults and early AD patients (Rodríguez-Aranda et al., 2016). It is also important to note that the degree of lateralization most likely depends on age, as functional neuroimaging studies on older participant have indicated a general reduction in hemispheric specialization in favor of more bilateral activation (Reuter-Lorenz et al., 2000; Cabeza, 2002). This age-related restructuring of the neural architecture has been posited to occur primarily by recruiting additional cortical areas to preserve performance and has been documented not only in VF tasks (Meinzer et al., 2012; La et al., 2016) and overt naming (Wierenga et al., 2008) but also in other cognitive functions such as the ventral visual system (Park et al., 2004) and the motor system (Carp et al., 2011). Furthermore, some investigators have speculated that the involvement of the right hemisphere in semantic fluency tasks may reflect the utilization of visuospatial mental imaging strategies for these tasks (Biesbroek et al., 2016; Gordon et al., 2018). Finally, it is important to note that only the models that included solely right-handed participants remained significant after multiple testing correction. This is discussed further in the limitations section.

Regarding the results, there are a number of null findings that need addressing. Possibly, the most relevant one is that no group-specific associations were seen for the MCI/AD patient group. This is in contrast with our previous results, as we have previously reported indications of a cumulative effect of WM pathology in the frontal areas on general cognitive functioning in AD patients specifically (Kaskikallio et al., 2019b). We have also found indications of similar group-specific cumulative effects of frontal and temporal WMH volumes on processing speed (Kaskikallio et al., 2019a, 2020). The results in the present study do not support the notion that WM pathology would have group-specific/cumulative effects on VF in MCI and AD patients, contrasting some earlier findings that have been reported (Kavcic et al., 2008; Chen et al., 2009). On the other hand, these studies contained more limited sample sizes and also focused on analyzing specific WM tracts, whereas the current study utilized volumetric WMH measurements of larger lobar areas. Despite the fact that our sample size was larger than those in previous studies, it could still be too small to detect smaller effects (see *Limitations*). Two other null findings should also be mentioned: (Feigin et al., 2003) no significant associations were found between VF tasks and left temporal WMH in the main analyses, although the region has been implicated heavily with semantic fluency tasks (Pantoni et al., 2007; Schmidt et al., 2019) no significant associations were seen between phonological fluency and WMH in any region. Possible reasons for these are discussed in section “Limitations and Recommendations.”

Although the cognitively healthy controls had, on average, higher VF performances that MCI/AD patients, the differences were not statistically significant. The difference was significant in the original sample, but regrettably, a number of participants had to be dropped due to inadequate imaging resolution for quantitative imaging analysis. Overall, the MCI/AD group utilized in the final sample has relatively good cognitive performance (a MMSE mean score of 24.16), which is also reflected as higher VF performance [compare with, for example, a study by Rinehardt et al. (2014), where VF performance of MCI and AD patients is on a notably lower level compared to the present study]. It should also be noted that since there were no significant differences in VF scores and WMH distributions between the subgroups, it is very likely that the AD patients included in the final sample (which formed the majority of the patient subgroup) were in relatively early phases of disease progression at the time of data collection.

Thus, although word finding difficulties can appear relatively early in AD and they are generally associated with VF scores (Farrell et al., 2014), these hindrances might not necessarily translate to significant deficits in VF for every patient. This implies that, in these cases, semantic information structures might still be relatively intact and accessible, although it is important to remember that VF performance is likely affected by a number of other components, including cognitive flexibility and strategy utilization, working memory, speed of processing and lexical retrieval, as well as basic linguistic abilities (Rinehardt et al., 2014; Whiteside et al., 2016; Schmidt et al., 2017; Gordon et al., 2018). From a methodological standpoint, it is worth noting that although VF tasks demand the retrieval of specific responses, they are less constrained than naming tasks for example (Gordon et al., 2018): If a certain word is not remembered in a VF task, a synonym can be used instead. In these cases, underlying vocabulary knowledge might be used to compensate for difficulties in word retrieval (Gordon et al., 2018). A related finding is that reading and writing habits, which are *per se* linked with vocabulary (Stanovich and Cunningham, 1992; Marulis and Neuman, 2010; Dylman et al., 2020), seem to be associated with VF performance in both cognitively healthy adults (Pawlowski et al., 2012) and patients with AD (Tessaro et al., 2020). In at least non-clinical participants, the effect seems to be even more stronger than education (Pawlowski et al., 2012). Another methodological issue to consider is the fact that differences exist regarding the cue content (i.e., object categories and letter cues used in tasks), timing (e.g., 60 vs. 90 s), and performance outcomes (e.g., correct words in total time limit, correct words in certain time intervals, latency between words, semantic clustering, etc.) of VF tasks in different studies. Variation in the background variables discussed here might also partly contribute to the non-significant subgroup differences in VF performance in the current study. Finally, regarding the effects of concomitant vascular pathology, it might be the case that AD-related disease progression must be at a more advanced stage before concomitant vascular pathology starts to have a cumulative effect on VF performances.

## Limitations and Recommendations

We acknowledge that the study has a number of limitations. First, the sample size (and thus the statistical power to detect the effects reported) is not optimal, as a notable number of participants had to be excluded due to insufficient MR image quality for quantification. *Post hoc* calculations concerning effect sizes seen in step 2 of hierarchical regression models indicate that the statistical power of the current total sample size is somewhat below the gold standard of 0.80 (0.60 for frontal bilateral WMH and 0.77 for bilateral parieto-occipital WMH). Thus, the relatively limited size of the final sample might have an effect on the statistical power to detect smaller effects especially in the patient subgroup and might also explain the null findings mentioned previously. Despite the relatively small sample size, our sample is still almost twice the size of previous published studies on the matter (Kavcic et al., 2008; Chen et al., 2009; Serra et al., 2010; Rodríguez-Aranda et al., 2016). Second, the diminished sample size also necessitated the merging of the MCI and AD patient subgroups, which, although being a fairly commonplace procedure in the literature, might not be the optimal solution. Third, since several hierarchical regression models have been run, the risk for family-wise Type I errors (detecting a false positive) is increased. We attempted to guard against false positives by using the Benjamini–Hochberg procedure. After correction, only analyses containing solely right-handed participants remained significant. It is possible that any confounding effects regarding language lateralization were nullified with the removal of left-handed/ambidextrous participants, leading to slightly stronger effects in the regression models. Regarding language lateralization, it is a well-known fact that right-handed participants are more homogeneous with regards to brain functions. As such, it is not surprising to have results change when non-right-handed participants are included or excluded from the analyses. Finally, the current study only utilized total performance scores for measuring VF. Complimentary methods for assessing VF, such as naming latency or semantic clustering, have also been developed.

Overall, further research is needed on the possible group-wise effects of WM pathology on VF in the MCI/AD continuum. As the current study contains a number of unexpected null results, we feel that it is important to keep in mind that a critical feature for research literature to be trustworthy is that “all studies with at least reasonable quality have been reported” (Cumming, 2013). This is especially important in order to minimize publication bias, i.e., the cherry picking of positive findings and the exclusion of null or ambivalent findings. As single studies are rarely final or conclusive, additional evidence is required in the form of replications, follow-up studies, and meta-analyses (Cumming, 2013). In the case of this study topic, future studies would do well to incorporate larger sample sizes and utilize heterogeneous measures for both imaging (e.g., diffusion tensor imaging of microstructural WM tract integrity and volumetric approximation of WMH) as well as for behavioral measurement (e.g., total performance scores, naming latency, semantic clustering). However, transparency about reporting the measures and calculations utilized in assessing VF should be an important goal, as differences here can lead to difficulties in interpreting and replicating the results. It would also be prudent to take into consideration the stage of disease progression in AD patients as well as measure/control background variables besides age and education, including linguistic abilities such as vocabulary and reading and writing habits. Due to the possibility of confounding effects, using solely right-handed participants might be recommendable.

## Conclusion

In conclusion, as has been shown elsewhere, frontal and parieto-occipital WMH seem to have an effect on semantic fluency. Elevated levels of WMH, as measured by volumetric imaging methods, seem to affect VF performances of both cognitively healthy adults and patients with MCI or AD, i.e., no additive effects of WMH in the patient group were found in this study. However, more research is needed on the possible group-wise effects of WM pathology on VF in the MCI/AD continuum, as the current study has a number of limitations, including the suboptimal statistical power of the current sample to detect the reported effects as well as the merging of the MCI/AD subgroups for analysis. We expect that future studies will elucidate the subject matter further: follow-up studies should aim to replicate the findings, incorporate larger sample sizes, utilize more heterogeneous imaging and behavioral measures, and account for background variables such as AD progression, vocabulary abilities, and reading and writing habits.

## Data Availability Statement

The raw data supporting the conclusions of this article will be made available by the authors, without undue reservation.

## Ethics Statement

The studies involving human participants were reviewed and approved by the Joint Ethical Committee of the University of Turku and Turku University City Hospital. The patients/participants provided their written informed consent to participate in this study.

## Author Contributions

AK performed the statistical analyses and wrote the initial draft. PG-N and MK helped in data analysis, interpretation, and manuscript drafting. JR organized the data collection, and together with MK and PG-N handled the project administration and supervision, as well as contributed to the conception of the study. TT and RP performed the original visual magnetic resonance analyses. JL and JK developed the methodology for the quantitative magnetic resonance imaging analysis and performed the analyses. JR, TT, RP, JL, and JK critically reviewed the manuscript. All authors have made substantial and direct contributions to the work, have approved the final version of the work, and agree to be accountable for all aspects of the work.

## Conflict of Interest

JK and JL were shareholders of Combinostics Ltd. and employed by the company Combinostics Oy. The remaining authors declare that the research was conducted in the absence of any commercial or financial relationships that could be construed as a potential conflict of interest.
